# The link between emotional health and organizational performance in the healthcare sector: a policy- and management-oriented perspective on psychological risks, organizational behavior, and service quality

**DOI:** 10.3389/fpsyg.2026.1799120

**Published:** 2026-07-03

**Authors:** Murat Başal, Ömer Faruk Şarkbay, Aslı Kaya

**Affiliations:** 1 Istanbul Gelisim University, Vocational School, İstanbul, Türkiye; 2 Faculty of Health, Istanbul Gelisim University, İstanbul, Türkiye; 3 Health Management, Faculty of Health Sciences, Istanbul Gelisim University, İstanbul, Türkiye

**Keywords:** emotional health, health systems governance, healthcare workforce well-being, organizational performance, service quality in healthcare

## Abstract

This perspective article examines the complex and increasingly consequential relationship between emotional health and organizational performance in the healthcare sector, positioning emotional well-being not as an individual psychological concern but as a strategic organizational and public health issue with direct implications for service quality, workforce sustainability, and system resilience. Against the backdrop of escalating workload pressures, workforce shortages, digital transformation, and rising patient expectations, healthcare systems worldwide are experiencing unprecedented levels of emotional strain among professionals, manifesting in burnout, emotional exhaustion, moral distress, and disengagement. Drawing on interdisciplinary insights from public health, organizational behavior, and health services management, this perspective argues that emotional health functions as a critical mediating mechanism through which organizational structures, leadership practices, and governance models shape both employee behavior and patient-facing outcomes. Rather than conceptualizing emotional health as a residual outcome of individual resilience or coping capacity, the article reframes it as a core input in healthcare service production, comparable in strategic importance to staffing levels, clinical competence, and technological infrastructure. Synthesizing recent empirical and conceptual research, the perspective highlights that emotional strain does not uniformly or linearly translate into reduced performance; instead, its effects are highly context-dependent and are mediated through behavioral, relational, and cultural mechanisms at multiple organizational levels. Emotionally strained healthcare professionals are more likely to disengage from discretionary behaviors such as empathic communication, proactive problem-solving, teamwork, and knowledge sharing, which are essential for maintaining service quality, patient safety, and continuity of care in complex clinical environments. Over time, this disengagement erodes psychological safety, weakens trust, and diminishes collective efficacy, thereby undermining organizational climate and long-term performance capacity rather than merely affecting short-term productivity indicators. From a public health perspective, the consequences of deteriorating emotional health extend beyond organizational boundaries, influencing treatment adherence, health equity, patient satisfaction, and public trust in health systems, particularly in primary care, chronic disease management, mental health services, and end-of-life care. The perspective further situates these dynamics within the context of contemporary health system transformations, including the rapid expansion of digital health technologies, artificial intelligence–supported decision-making, and performance-based governance models. While such innovations offer opportunities to reduce cognitive overload and improve efficiency, the article critically notes that algorithmic management, intensified performance monitoring, and reduced relational time may exacerbate emotional alienation if emotional labor and psychological safety are not explicitly addressed in system design and governance. A central contribution of this perspective lies in its emphasis on the moderating role of organizational support and institutional culture. Evidence from recent organizational research consistently demonstrates that perceived organizational support, supportive leadership behaviors, participatory governance structures, and cultures that legitimize emotional vulnerability significantly buffer the negative effects of emotional strain on outcomes such as turnover intention, disengagement, and service quality. Conversely, performance-driven cultures that normalize emotional suppression and individualize responsibility for coping risk amplifying psychological risks, silent withdrawal, and moral injury, even when formal well-being initiatives are present. In critically assessing the strengths and limitations of current policy and management approaches, the article identifies a key strength in the growing recognition of workforce well-being as a system-level concern within international health policy discourse, alongside a persistent weakness in the dominance of outcome-focused performance metrics that neglect emotional labor as a process variable. This misalignment creates both risks and opportunities: while failure to integrate emotional health into governance frameworks threatens workforce sustainability and public trust, embedding emotional health indicators into quality management systems, accreditation processes, and performance dashboards offers a powerful opportunity for earlier risk detection, preventive intervention, and more equitable and resilient health system design. Translating these insights into action, the perspective outlines a set of evidence-informed policy and management implications that emphasize the need to move beyond fragmented, individual-level interventions toward integrated organizational redesign. Key recommendations include incorporating emotional health metrics—such as burnout, emotional exhaustion, moral distress, and psychological safety—into organizational performance frameworks; prioritizing workload regulation, staffing adequacy, and emotionally sustainable service models; strengthening leadership accountability for emotional climates; embedding emotional health considerations into workforce planning and retention strategies; and fostering organizational cultures that normalize emotional support and collective responsibility for well-being. Importantly, the article underscores that leadership capacity to recognize and respond to emotional strain constitutes a core managerial competency rather than a peripheral “soft skill,” with direct implications for service quality and organizational legitimacy. From a future research directions standpoint, the perspective calls for a paradigmatic shift in research design and focus. It highlights the limitations of cross-sectional, individual-level studies and advocates for multi-level, longitudinal, and comparative research capable of capturing how emotional health dynamics evolve over time across individuals, teams, organizations, and health systems, and how they interact with governance reforms, digital innovations, and labor market conditions. Comparative policy research across different health system models is identified as a particularly promising avenue for understanding how institutional design influences emotional health trajectories and performance resilience. At the same time, the article cautions against the uncritical adoption of emotional health metrics, emphasizing the ethical and methodological challenges associated with measurement, data use, and potential stigmatization if emotional indicators are instrumentalized without supportive governance. Overall, this perspective advances an integrative framework that positions emotional health as a central pillar of organizational performance, service quality, and public health outcomes in the healthcare sector. By bridging disciplinary silos and aligning research agendas with the realities of policy-making and management practice, the article offers a compelling case for emotionally informed governance as both a scientific priority and a practical necessity. In doing so, it provides clear key takeaways for policy and practice: emotional health must be treated as a strategic performance input; organizational context matters as much as individual capacity; leadership and culture are decisive leverage points; and sustainable healthcare systems depend on governance models that recognize, protect, and actively cultivate the emotional foundations of care work.

## 1 Introduction: emotional health as a public health and management priority

Healthcare systems are facing a convergence of structural pressures that extend far beyond clinical capacity and technological infrastructure. Workforce shortages, increasing service demand, aging populations, and recurrent public health crises have placed healthcare professionals under sustained emotional strain. In recent years, international health organizations and policy bodies have increasingly recognized that the emotional health of healthcare workers is not only an occupational issue but also a public health and governance concern, directly affecting service quality, patient safety, and system sustainability (World Health Organization, 2022; OECD, 2023a,b).

Empirical evidence accumulated over the past 5 years demonstrates a consistent rise in burnout, emotional exhaustion, anxiety, and moral distress among healthcare professionals across countries and care settings. Importantly, these emotional health challenges are no longer confined to individual well-being outcomes. Instead, they manifest at the organizational level through reduced service quality, increased turnover intention, weakened patient trust, and declining institutional resilience. Despite this growing evidence, healthcare policy and management frameworks continue to treat emotional health largely as a secondary or reactive issue, addressed through individual-level interventions rather than organizational redesign.

This perspective argues that such an approach is no longer sufficient. Emotional health must be reframed as a core organizational performance determinant and an integral component of health system governance. From a public health and health services management standpoint, emotional health represents a strategic resource whose deterioration generates systemic risk. Conversely, when actively supported, it functions as a protective mechanism that sustains service quality and organizational continuity under high-pressure conditions.

## 2 From individual distress to organizational risk accumulation

Traditional approaches to healthcare workforce well-being have predominantly emphasized individual coping, resilience training, and stress management programs. While these interventions may offer short-term relief, recent research suggests that they often fail to address the structural and organizational origins of emotional strain. Studies conducted during and after the COVID-19 pandemic clearly demonstrate that emotional exhaustion and burnout are strongly associated with workload intensity, staffing shortages, role ambiguity, and emotionally demanding service interactions—factors largely outside individual control (Bakker et al., 2023; West et al., 2020).

From a systems perspective, emotional health problems should be understood as organizational risk accumulators. Emotional exhaustion impairs cognitive functioning, ethical sensitivity, and interpersonal communication—capabilities that are critical in healthcare delivery. When such impairments become widespread within an organization, they increase the likelihood of medical errors, weaken coordination across teams, and undermine patient-provider relationships. These dynamics transform emotional health from a private concern into a collective vulnerability with measurable performance consequences.

Recent health services research underscores that hospitals and healthcare organizations with higher levels of workforce burnout exhibit poorer patient safety climates, lower patient satisfaction scores, and higher rates of adverse events. These findings suggest that emotional health operates as an upstream determinant of service outcomes, warranting attention at the policy and management levels rather than being relegated to occupational health units alone.

## 3 Organizational behavior mechanisms linking emotional health to performance

Organizational behavior theories provide a critical lens for understanding how emotional health is translated into individual and collective performance outcomes, particularly in emotionally intensive contexts such as healthcare. Within this body of work, the job demands-resources (JD-R) model remains one of the most influential explanatory frameworks, suggesting that persistent job demands—such as emotional labor, moral distress, time pressure, patient acuity, and exposure to suffering—consume psychological and energetic resources and lead to emotional exhaustion when not counterbalanced by adequate organizational resources (Demerouti et al., 2021; Bakker and de Vries, 2021). Contemporary research, however, increasingly challenges the assumption that emotional exhaustion affects performance in a direct or uniform manner. Instead, scholars emphasize that its effects are mediated through behavioral, relational, and cultural mechanisms embedded within the organization. Empirical studies demonstrate that emotionally exhausted healthcare professionals tend to withdraw from discretionary and prosocial behaviors, including empathic patient engagement, proactive communication, knowledge sharing, and collaborative problem-solving, which are critical for service quality and safety in complex care environments (Montani et al., 2024; Huertas-Valdivia et al., 2023). Over time, this behavioral disengagement undermines psychological safety, weakens interpersonal trust, and erodes collective efficacy, thereby diminishing organizational climate and long-term performance capacity rather than merely reducing short-term productivity. From a managerial and governance perspective, these findings underscore that emotional health is deeply embedded in leadership styles, organizational culture, and structural design. Leadership approaches that prioritize efficiency, standardization, and performance metrics without recognizing emotional demands may unintentionally intensify burnout risks, normalize emotional suppression, and exacerbate silent withdrawal behaviors. In contrast, supportive and ethical leadership, participatory decision-making, fair workload allocation, and recognition of emotional labor function as critical organizational resources that buffer emotional strain and sustain adaptive performance (West et al., 2023; Montani et al., 2017; Shanafelt and Noseworthy, 2024). Recent studies further extend this discussion by situating emotional health within the context of digital transformation and artificial intelligence–enabled work systems. While AI-based clinical decision support, automation, and workflow optimization offer significant opportunities to reduce cognitive overload and routine task pressure, emerging evidence also highlights potential negative consequences, such as algorithmic surveillance, perceived loss of professional autonomy, accelerated work pace, and reduced human interaction, all of which may amplify emotional alienation if not governed responsibly (Kellogg et al., 2020; 2024). Thus, a critical strength of current organizational behavior research lies in its recognition of emotional health as a strategic organizational asset rather than an individual vulnerability; a key weakness remains the persistence of managerial models that treat emotions as peripheral or private concerns. Importantly, the literature identifies a substantial opportunity for organizations that integrate emotional health into governance frameworks, leadership development, and human-centered digital strategies to achieve sustainable performance, resilience, and trust. Conversely, the primary threat arises when emotional health is neglected, leading to cumulative disengagement, service quality erosion, and long-term reputational and institutional damage.

## 4 Emotional health and service quality: a public health lens

From a public health perspective, service quality in healthcare cannot be meaningfully separated from the emotional health and emotional labor of the workforce. Unlike standardized or technology-mediated services, healthcare delivery is inherently relational and moral in nature, requiring sustained empathy, attentiveness, compassion, and situational judgment in conditions of uncertainty and vulnerability. A growing body of evidence demonstrates that these relational competencies are not merely individual traits but are dynamically shaped by the emotional well-being of healthcare professionals and the organizational environments in which they operate (Tronto, 2013; Maben et al., 2020). When emotional health deteriorates due to chronic workload pressure, moral distress, or insufficient organizational support, service encounters tend to shift toward transactional and task-oriented interactions, resulting in diminished patient engagement, reduced perceived service quality, and lower satisfaction levels (Dyrbye et al., 2023). Importantly, public health research highlights that the consequences of emotionally degraded service quality extend far beyond individual patient experiences. Poor relational quality in care has been linked to lower treatment adherence, delayed help-seeking behavior, increased use of emergency services, and the reinforcement of existing health inequalities, particularly among older adults, individuals with chronic conditions, and socioeconomically disadvantaged populations (Street et al., 2022; World Health Organization, 2023). Recent empirical studies further indicate that patients are highly sensitive to providers’ emotional availability and perceived presence, especially in primary care, chronic disease management, mental health services, and end-of-life care, where trust and continuity are central determinants of outcomes (Rathert et al., 2023; Beach et al., 2024). In this context, emotional exhaustion among healthcare staff emerges as a population-level risk factor rather than a purely organizational issue, with implications for public trust in health systems and the legitimacy of health governance itself. While digital health technologies and artificial intelligence–supported service models offer opportunities to enhance efficiency and standardization, the literature cautions that these innovations may unintentionally intensify emotional distancing if relational dimensions of care are marginalized, thereby weakening service quality from a public health standpoint (Greenhalgh et al., 2023). A key strength of current policy-oriented research lies in its growing recognition that workforce emotional health constitutes a foundational input in healthcare service production; however, a persistent weakness remains the dominance of outcome-focused quality metrics that neglect emotional labor as a process variable. Consequently, scholars and international health organizations increasingly advocate for the integration of emotional health and psychosocial work environment indicators into service quality monitoring, accreditation systems, and value-based care frameworks (OECD, 2023a,b). Such an approach represents a significant opportunity to shift health system governance toward more process-sensitive, equitable, and sustainable models of quality improvement, while failure to address emotional health systematically poses a substantial threat in the form of declining care quality, workforce attrition, and long-term erosion of public confidence in healthcare institutions.

## 5 The moderating role of organizational support and institutional culture

Recent organizational and occupational health research consistently demonstrates that the detrimental effects of emotional strain on employee behavior and performance are highly contingent upon contextual and institutional factors rather than being inevitable or uniform. Among these factors, perceived organizational support (POS)—defined as employees’ generalized belief that their organization values their contributions and genuinely cares about their well-being—has emerged as one of the most robust buffering mechanisms within high-strain work environments such as healthcare (Eisenberger et al., 1986; Kurtessis et al., 2017). Meta-analytic and longitudinal evidence published in recent years indicates that high levels of POS significantly attenuate the relationship between emotional exhaustion and adverse outcomes, including turnover intention, work disengagement, cynicism, and declines in service quality, while simultaneously strengthening affective commitment and discretionary performance behaviors (Kurtessis et al., 2017; Lee and Peccei, 2023). Importantly, contemporary studies emphasize that organizational support operates not merely as a compensatory resource but as a symbolic signal that shapes how emotional demands are interpreted and legitimized within the workplace. In healthcare organizations where emotional strain is acknowledged as an inherent and collective dimension of caring labor—rather than stigmatized as a sign of individual weakness—employees are more likely to engage in help-seeking, peer support, and open emotional communication, thereby transforming emotional health into a shared organizational responsibility embedded in institutional culture (Maben et al., 2020; West et al., 2023). Such cultures foster psychological safety and normalize vulnerability, which has been shown to enhance team learning, adaptive performance, and collective resilience in complex clinical settings. Conversely, cultures characterized by implicit expectations of emotional invulnerability, performance-at-all-costs metrics, and moralized notions of professional sacrifice may unintentionally intensify emotional suppression, silent withdrawal, and moral distress, even when formal support mechanisms are nominally present. From a strengths perspective, organizations that integrate supportive leadership, fair and transparent decision-making, and emotionally intelligent governance structures are better positioned to sustain workforce well-being and service quality simultaneously; however, a persistent weakness in many health systems lies in the misalignment between espoused values of support and actual resource allocation practices, particularly regarding staffing ratios, workload distribution, and time for relational work. Emerging opportunities are increasingly identified in policy-oriented research, which calls for embedding emotional health indicators into organizational performance dashboards, leadership accountability frameworks, and accreditation standards, thereby shifting governance from individual resilience paradigms toward system-level responsibility (OECD, 2023a,b; World Health Organization, 2023). At the same time, a key threat highlighted in recent literature concerns the rapid expansion of digitally mediated performance monitoring and algorithmic management, which—if not accompanied by strong cultures of support and trust—may undermine POS by signaling control rather than care. Taken together, these findings align closely with the frontiers tradition of integrative and systems-oriented scholarship, underscoring that emotional health outcomes in healthcare are not solely a function of personal coping capacities but are fundamentally shaped by organizational design choices, institutional culture, and governance decisions that either buffer or amplify emotional strain.

## 6 Implications for health policy and management practice

Reframing emotional health as a systemic organizational and public health issue rather than an individual coping problem carries far-reaching implications for health policy and management practice, particularly in the context of increasingly strained and digitally transformed health systems. First, the literature strongly suggests that workforce well-being strategies must move beyond fragmented, short-term interventions—such as mindfulness training or individual resilience workshops—toward integrated and institutionally embedded policies that align emotional health with human resource planning, quality management systems, and organizational governance frameworks (West et al., 2023; Dyrbye et al., 2023). When emotional health considerations are incorporated into staffing models, workload design, performance evaluation criteria, and leadership accountability mechanisms, organizations are better positioned to address the structural drivers of emotional strain rather than merely its symptoms. A key strength of such an approach lies in its capacity to enhance sustainability at both workforce and system levels; however, a persistent weakness remains the operationalization challenge, as emotional health indicators are often perceived as intangible, context-dependent, and difficult to standardize across institutions. Second, at the policy level, there is growing consensus among international organizations and health system scholars that emotional health metrics should be integrated into system performance dashboards alongside traditional indicators of efficiency, access, and clinical outcomes (OECD, 2023a,b; World Health Organization, 2023). Empirical evidence increasingly demonstrates that workforce sustainability is inseparable from service sustainability, and that early warning signs of emotional exhaustion, moral distress, and disengagement can serve as leading indicators of declining service quality, safety risks, and organizational fragility (Shanafelt and Noseworthy, 2017). The inclusion of such metrics offers a significant opportunity for more proactive, preventive governance; nevertheless, it also raises critical concerns regarding data misuse, surveillance, and the potential stigmatization of organizations or professional groups if emotional health data are not interpreted within a supportive and developmental policy framework. Third, implications for management education and leadership development are particularly salient. Recent studies underscore that healthcare leaders play a decisive role in shaping emotional climates through everyday practices related to communication, prioritization, and symbolic signaling, and that leadership capacity to recognize, legitimize, and respond to emotional strain constitutes a core managerial competence rather than a “soft” or ancillary skill (Edmondson and Bransby, 2023; Montgomery et al., 2024). Integrating emotional labor, ethical stress, and psychological safety into formal leadership curricula represents a major opportunity to recalibrate managerial norms toward more humane and resilient models of healthcare delivery. At the same time, the rapid expansion of algorithmic management, performance monitoring technologies, and value-based payment systems poses a potential threat if emotional health considerations are not explicitly embedded into their design and governance. From a future research perspective, frontiers-oriented scholarship would benefit from longitudinal and multi-level studies examining how emotional health indicators interact with policy reforms, digital health innovations, and leadership development initiatives over time, as well as comparative research exploring cross-national differences in how health systems institutionalize emotional health within governance and accreditation regimes. Such work is essential for advancing a more integrated, evidence-based, and ethically grounded approach to health policy and management in an era of escalating workforce vulnerability and system complexity.

## 7 Toward an integrative research and action agenda

This Perspective underscores the urgent need for a more integrative research and action agenda that systematically bridges public health, organizational behavior, and health services management to better understand and address emotional health in healthcare systems. Despite growing recognition of workforce emotional well-being as a determinant of service quality and system resilience, much of the existing literature remains fragmented, cross-sectional, and predominantly focused on individual-level outcomes, thereby limiting its capacity to inform structural and policy-level interventions. Future research would substantially benefit from multi-level and longitudinal designs capable of capturing how emotional health dynamics unfold over time across individual, team, organizational, and system levels, and how these dynamics interact with changing governance arrangements, workforce policies, and digital transformations (West et al., 2023; Dyrbye et al., 2023). Comparative and cross-national policy research represents a particularly promising opportunity, as differing health system governance models—such as centralized versus decentralized systems, market-oriented versus solidarity-based financing, and varying degrees of managerial autonomy—offer natural experiments for examining how institutional design shapes emotional health trajectories and performance resilience among healthcare workers (OECD, 2023a,b; World Health Organization, 2023). At the same time, a critical weakness in the current research landscape lies in the persistent overemphasis on individual coping mechanisms and resilience training, which, while valuable, risk obscuring the structural and organizational drivers of emotional strain. Emerging evidence increasingly points to the effectiveness of organizational-level interventions, including workload redesign, staffing adequacy, participatory governance mechanisms, psychologically safe team practices, and supportive leadership structures, in producing more sustainable improvements in emotional health and performance outcomes than isolated individual-focused interventions (Edmondson and Bransby, 2023; Montgomery et al., 2024). From a strengths perspective, the growing availability of administrative data, employee experience surveys, and digital work analytics creates new methodological opportunities for integrating emotional health indicators into routine organizational and system-level evaluation; however, these developments also pose ethical and methodological challenges related to surveillance, data interpretation, and the potential instrumentalization of emotional metrics. Looking forward, frontiers-oriented scholarship is well positioned to advance action-oriented research that not only explains emotional health phenomena but also evaluates the design, implementation, and unintended consequences of organizational and policy interventions in real-world healthcare settings. Such an agenda would align research more closely with the practical realities of healthcare management and policy decision-making, while also addressing a critical threat identified in the literature: the risk that failure to move beyond individual-level explanations will perpetuate workforce vulnerability, undermine trust, and weaken the long-term sustainability of health systems. Collectively, these directions call for a paradigmatic shift toward emotionally informed governance and system-level accountability, positioning emotional health as a central pillar of both research inquiry and practical action in contemporary healthcare.

## 8 Policy and management implications: translating emotional health into action

Translating emotional health from an abstract concern into actionable policy and management practice requires a deliberate shift toward evidence-informed governance that recognizes emotional well-being as a core determinant of healthcare system performance and resilience. Accumulating research suggests that integrating workforce emotional health indicators—such as burnout, emotional exhaustion, moral distress, and psychological safety—into organizational performance frameworks and accreditation systems enables earlier identification of systemic risk and supports proactive, preventive intervention rather than reactive crisis management (Dyrbye et al., 2023; OECD, 2023a,b). A key strength of this approach lies in reframing emotional health as a legitimate performance input rather than a residual outcome; however, a persistent challenge concerns measurement validity and the risk that emotional metrics may be reduced to compliance-oriented reporting if not embedded within supportive learning systems. Evidence further indicates that while individualized resilience and well-being programs can provide short-term benefits, their effectiveness remains limited when organizational drivers of strain—such as excessive workload, staffing shortages, role ambiguity, and emotionally unsustainable service models—are left unaddressed (Montgomery et al., 2024). Consequently, policy and management strategies that prioritize organizational redesign, including workload regulation, staffing adequacy, participatory decision-making, and continuity-oriented care models, represent a critical opportunity to address root causes of psychological strain rather than its symptoms. Leadership accountability emerges as another pivotal leverage point: empirical studies consistently show that leadership behaviors and symbolic actions strongly shape emotional climates, psychological safety, and help-seeking norms, underscoring that emotional labor management and supportive supervision constitute core managerial competencies rather than discretionary “soft skills” (Edmondson and Bransby, 2023; West et al., 2023). Embedding these competencies into leadership development, selection, and evaluation frameworks strengthens alignment between espoused organizational values and everyday managerial practice, although resistance may arise in performance-driven cultures that continue to privilege efficiency over relational work. At the system level, national and regional workforce planning strategies increasingly recognize emotional health as a determinant of retention, productivity, and service continuity, particularly amid global shortages of healthcare professionals and rising demand complexity (World Health Organization, 2023). Aligning workforce planning with emotional health considerations offers a significant opportunity to enhance system resilience, yet failure to do so poses a clear threat in the form of accelerated turnover, skill erosion, and declining public trust. Finally, sustained progress depends on investment in longitudinal and multi-level research capable of examining how emotional health dynamics evolve over time and across individual, organizational, and policy levels, as well as how emerging governance instruments—such as value-based payment systems and digitally mediated performance monitoring—interact with emotional well-being. From a frontiers-oriented perspective, such research is essential not only for refining theory but also for generating actionable insights that inform ethically grounded, emotionally informed governance models, thereby translating emotional health into a shared responsibility and a strategic pillar of health policy and management practice.

## 9 Conclusion: emotional health as a governance imperative

Emotional health in the healthcare workforce can no longer be regarded as a peripheral, individual, or discretionary issue addressed solely through occupational health initiatives or short-term well-being programs. Accumulating evidence over the past 5 years clearly demonstrates that emotional health constitutes a foundational determinant of organizational performance, service quality, and public trust in health systems. Burnout, emotional exhaustion, and moral distress among healthcare professionals have repeatedly been linked to compromised patient safety, reduced quality of care, workforce instability, and declining system resilience (West et al., 2020; World Health Organization, 2022; OECD, 2023a,b).

This perspective advances the argument that emotional health should be repositioned from an individual pathology framework toward an organizational and governance-oriented paradigm. While prior research has made significant progress in documenting the prevalence and consequences of burnout, much of the literature continues to conceptualize emotional health as an outcome variable rather than as a strategic organizational resource. As a result, policy responses often remain reactive, fragmented, and insufficiently embedded within broader health system governance structures (Demerouti et al., 2023; Belita et al., 2025).

By contrast, viewing emotional health through a governance lens reveals its role as a system-level capacity that enables healthcare organizations to absorb shocks, adapt to crises, and sustain performance under conditions of chronic pressure. Recent comparative studies suggest that healthcare systems investing in supportive organizational cultures, participatory leadership, and workforce-centered governance exhibit greater resilience and continuity during periods of disruption, including public health emergencies (OECD, 2023a,b). These findings align with organizational behavior research demonstrating that emotional health is deeply intertwined with collective sensemaking, coordination quality, and ethical decision-making—core processes underpinning high-performing healthcare organizations (Bakker et al., 2023).

Importantly, this perspective builds on and extends recent calls in public health and health services research to integrate workforce well-being into performance and quality frameworks. While initiatives such as the WHO’s Mental Health at Work policy brief emphasize the moral and human rights dimensions of workforce mental health, the present analysis underscores that emotional health is also a governance imperative with tangible implications for service delivery and system sustainability (World Health Organization, 2022). Similarly, health services studies increasingly highlight that neglecting emotional health generates hidden organizational costs, including turnover, absenteeism, and diminished patient trust, which ultimately undermine public health goals (West et al., 2020; Kurtessis et al., 2023).

From a policy and management perspective, the implications are clear. Emotional health must be systematically embedded in health system governance through workforce planning, quality management, leadership accountability, and performance monitoring. Treating emotional health as an organizational resource necessitates moving beyond individual resilience narratives toward structural and cultural interventions that address workload design, emotional labor demands, and psychological safety. Such an approach aligns with emerging evidence that organizational-level interventions—rather than solely individual-focused programs—yield more sustainable improvements in both workforce well-being and service outcomes (Demerouti et al., 2021; Belita et al., 2025).

In conclusion, addressing emotional health at the organizational and policy levels is not merely a moral obligation toward healthcare workers; it is a prerequisite for sustainable healthcare performance in the 21st century. As healthcare systems confront escalating demand, workforce shortages, and recurrent crises, emotional health must be recognized as a central pillar of governance, alongside financing, infrastructure, and technology. Future research and policy agendas that fail to incorporate this perspective risk perpetuating a cycle of workforce depletion and service deterioration. Conversely, those that position emotional health as a strategic governance priority will be better equipped to deliver high-quality, equitable, and resilient healthcare systems.

Looking ahead, the challenge for healthcare systems is no longer whether emotional health should be addressed, but how decisively and systematically it will be governed. As healthcare organizations confront persistent workforce shortages, escalating service demand, and increasing public scrutiny, emotional health will increasingly function as a critical fault line separating resilient systems from fragile ones. Future health policies that continue to externalize emotional health as an individual responsibility risk reinforcing cycles of burnout, disengagement, and service deterioration.

By contrast, health systems that proactively integrate emotional health into governance structures—through leadership accountability, organizational design, and performance evaluation—will be better positioned to sustain high-quality, equitable, and patient-centered care. From a forward-looking perspective, emotional health should be treated as a leading indicator of system performance, signaling emerging organizational risks before they manifest as safety incidents, workforce attrition, or public trust erosion. Embedding emotional health within health services governance is therefore not a peripheral reform but a strategic investment in the long-term viability of healthcare systems. Future research, policy experimentation, and managerial innovation should converge around this imperative, ensuring that emotional health becomes a cornerstone of sustainable healthcare governance rather than an afterthought.

## Data Availability

The original contributions presented in the study are included in the article/supplementary material, further inquiries can be directed to the corresponding author.
